# *Fusobacterium nucleatum* Facilitates M2 Macrophage Polarization and Colorectal Carcinoma Progression by Activating TLR4/NF-*κ*B/S100A9 Cascade

**DOI:** 10.3389/fimmu.2021.658681

**Published:** 2021-05-21

**Authors:** Lijun Hu, Yan Liu, Xuehua Kong, Rui Wu, Qi Peng, Yan Zhang, Lan Zhou, Liang Duan

**Affiliations:** ^1^ Key Laboratory of Laboratory Medical Diagnostics, Ministry of Education, Department of Laboratory Medicine, Chongqing Medical University, Chongqing, China; ^2^ Department of Laboratory Medicine, The First Affiliated Hospital of Chongqing Medical University, Chongqing, China; ^3^ Department of Laboratory Medicine, The Second Affiliated Hospital of Chongqing Medical University, Chongqing, China

**Keywords:** *Fusobacterium nucleatum*, colorectal carcinoma, macrophage, S100A9, TLR4

## Abstract

*Fusobacterium nucleatum* (*Fn*) has been considered as a significant contributor in promoting colorectal carcinoma (CRC) development by suppressing host anti-tumor immunity. Recent studies demonstrated that the aggregation of M2 macrophage (M*φ*) was involved in CRC progress driven by *Fn* infection. However, the underlying molecular mechanisms are poorly characterized. Here, we investigated the role of *Fn* in M*φ* polarization as well as its effect on CRC malignancy. *Fn* infection facilitated differentiation of M*φ* into the M2-like M*φ* phenotype by *in vitro* study. Histological observation from *Fn*-positive CRC tissues confirmed the abundance of tumor-infiltrating M2-like M*φ*. *Fn*-induced M2-like M*φ* polarization was weakened once inhibiting a highly expressed damage-associated molecular pattern (DAMP) molecule S100A9 mainly derived from *Fn*-challenged M*φ* and CRC cells. In addition, *Fn*-challenged M2-like M*φ* conferred CRC cells a more malignant phenotype, showing stronger proliferation and migration characteristics *in vitro* and significantly enhanced tumor growth *in vivo*, all of which were partially inhibited when S100A9 was lost. Mechanistic studies further demonstrated that activation of TLR4/NF-*κ*B signaling pathway mediated *Fn*-induced S100A9 expression and subsequent M2-like M*φ* activation. Collectively, these findings indicate that elevated S100A9 in *Fn*-infected CRC microenvironment participates in M2-like M*φ* polarization, thereby facilitating CRC malignancy. Furthermore, targeting TLR4/NF-*κ*B/S100A9 cascade may serve as promising immunotherapeutic strategy for *Fn*-associated CRC.

## Introduction

Colorectal cancer (CRC) is a common digestive system neoplasm, ranking the third most common cancer worldwide and the fourth most frequent cause of cancer death following lung, liver, and stomach cancer (1). Over the past two decades, although early screening and detection have significantly reduced the incidence and mortality of adults aged 50 years with CRC, CRC still maintains a steadily rising trend in younger individuals (2, 3). Recently, accumulating evidence by metagenome-wide association studies indicated an association between gut microbiota dysbiosis and CRC, and enteropathogenic microorganism plays a role in shaping the inflammatory environment and promoting tumor initiation and progression (4, 5). Nevertheless, some key pathogenic bacteria as well as their promoting effect on CRC still need to be further clarified.

*Fusobacterium nucleatum* (*Fn*), a Gram-negative oral commensal anaerobe, has been found highly enriched in inflammatory bowel disease (IBD) and was regarded as a potential risk indicator of inflammation-associated CRC (6, 7). Colonization of *Fn* contributed to carcinogenesis and was associated with the poor prognosis and short-term survival of clinical CRC patients (8–10). Previous studies have reported a direct regulating effect of *Fn* on human multi-type immunocyte, including lymphocytes and NK cells, facilitating periodontitis severity (11, 12). In CRC, involvement of *Fn* in mediating tumor immune escape occurrence by regulating NK cell cytotoxicity and tumor-infiltrating T lymphocyte cell activities has also been reported (13). Recently, *Fn* infection was reported to increase tumor-associated macrophage (TAM) infiltration and participated in mediating M2-M*φ* polarization, facilitating CRC progression, suggesting a crucial anti-tumor effect of tumor-associated macrophages elicited by *Fn* infection (8). Nevertheless, detailed mechanistic investigations about the presence and activation state of M*φ* within *Fn*-infected CRC are scarce.

Inflammatory mediator S100A9 was secreted as a damage-associated molecular pattern (DAMP) in the tumor microenvironment (TME) of many inflammatory tumors, including gastric cancer, prostate cancer, and liver cancer (14–16). By binding its main TLR4 and RAGE receptors, S100A9 participated in regulating inflammatory-immune response and tumor progression (17). We previously found that elevated S100A9 in TME promotes CRC development by directly acting on the CRC cells and indirectly regulating myeloid-derived suppressor cell (MDSC)-mediated T cell immunosuppression (18, 19). However, the regulatory mechanism of high levels of S100A9 in TME and whether S100A9 acts on other types of cells in the microenvironment remain elusive.

Recently, S100A9 has been reported to be regulated by some pathogen infection and exerted a promoting role in several types of cancer such as Hepatitis B virus (HBV)-related hepatocellular carcinoma (HCC) and HPV-infected skin lesions and cancer (16, 20). It is well-established that TLR4, an important receptor for pathogen-associated molecular pattern (PAMP), mediated downstream NF-κB activation in Fn-infected CRC cells, and was involved in the oncogenic cascade of CRC (21). S100A9, a newly identified NF-*κ*B target gene identified in multi-type of cancer, was also demonstrated to be involved in myeloid cell differentiation into M2-like M*φ* in CRC (22, 23). Thus, these observations led us to explore whether gut microbe *Fn* is involved in M*φ* activation by inducing the NF-*κ*B/S100A9 cascade in a TLR4-dependent manner in *Fn*-infected CRC.

In the current study, we explored the impact of *Fn* on M*φ* polarization in the TME of CRC and the consequences on tumor growth in subcutaneous tumor formation in nude mice. We found that activation of TLR4-mediated NF-*κ*B/S100A9 signaling pathways is involved in *Fn*-induced M2-like M*φ* polarization, which further promotes the progression of CRC. Our findings highlight the significance of S100A9 in regulating M2-like M*φ* polarization in *Fn*-infected CRC and implicate that S100A9 may serve as a potential intervention target in *Fn*-associated CRC patients.

## Materials and Methods

### Bacterial Strains and Culture Methods

*Fn* strain ATCC 25586 was purchased from the American Type Culture Collection (ATCC; Manassas, VA), and bacteria were cultured in brain heart infusion (BHI) containing hemin, K_2_HPO_4_, Vitamin K1, and L-Cysteine under anaerobic conditions at 37°C as previously described (24). *E. coli* was cultured for 24 h in Luria–Bertani (LB) medium overnight at 37°C in an orbital shaker incubator. Both bacteria were collected by 4°C centrifugation at 6,000×g for 8 min, and bacterial suspension was prepared after washing three times with sterile PBS. Turbidimetry was used to determine the turbidity of bacterial suspensions and adjust its concentration to 1 × 10^8^ CFU/ml. The following experiments were all processed at a multiplicity of infection (MOI) of 100:1.

### Cell Line Culture and M*φ* Differentiation of THP-1

Human CRC cell lines HCT116 and SW480 and human monocyte THP-1 were presented by the Chongqing Key Laboratory of Molecular Oncology and Epigenetics, the First Affiliated Hospital of Chongqing Medical University. HCT116 and SW480 cells were cultured in Dulbecco’s modified Eagle’s medium (DMEM), and THP-1 cells were cultured in Roswell Park Memorial Institute (RPMI)-1640 medium. Both media were supplemented with 10% fetal bovine serum (FBS) (Gibco, USA). The final concentrations of penicillin and streptomycin (Hyclone, USA) in these two media were 100 U/ml and 100 µg/ml, respectively. Cell culture was maintained at 37°C in an incubator containing 5% CO_2_, 95% air.

THP-1 cells were treated with PMA (50 ng/ml) (Sigma, USA) for 24 h to obtain differentiated M*φ* as demonstrated previously (25). Successfully differentiated M*φ*s were washed twice with PBS and incubated in fresh medium to be used for the following experiments.

### Clinical Specimens

CRC and matching distal normal tissues were collected from 16 patients who had undergone colorectal resection at the First Affiliated Hospital of the Chongqing Medical University. The clinicopathological data of the subjects including gender, age, Dukes staging, and lymphatic metastasis at initial diagnosis are shown in **Table S1**. The patients received no chemotherapy, hormonal therapy, or radiotherapy before surgery, and written informed consent was received from all participants. This study was approved by the Ethics Committee of Chongqing Medical University (protocol number 2012-19).

### Preparation of the Recombinant Proteins

Recombinant S100A9 (rS100A9) proteins used in this study have been described previously (18). Briefly, the pGST-moluc and pGST-moluc-S100A9 were transformed into BL21 bacteria following the instructions for calcium chloride transformation. Isopropylthio-*β*-D-galactoside was used to induce the expression of GST and GST-S100A9 proteins. The supernatant of sonicated bacteria was collected, and then incubated with glutathione-sepharose 4B beads. Then, rS100A9 and control GST on the beads were eluted using elution buffer with reduced glutathione. Finally, the two recombinant proteins were filtered with 0.22 μm membrane and stored at −80°C.

### Preparation of Conditioned Medium From Cells With Various Treatments

M*φ* or CRC cells were seeded in six-well plates and then infected with bacteria. After 48 h, the supernatant was harvested and clarified by centrifugation to remove the bacteria, cells and their debris, and then stored at −80°C refrigerator for later use.

M*φ* or CRC cells were transfected with non-specific siRNA (siNC) or S100A9-siRNA (siS100A9) (GenePharma, China) (**Table 1**) at a final concentration of 100 pM with lipofectamine 2000 reagent (Invitrogen, USA) in serum-free medium. The medium was replaced with fresh medium containing 10% FBS after 6 h of transfection and *Fn* was added to each group. The supernatant was collected after 48 h in the same manner as before.

**Table 1:** The primers and siRNA used in this study.

Genes		Sequences
*GAPDH*	Forward primer:	CAGCGACACCCACTCCTC
	Reverse primer:	TGAGGTCCACCACCCTGT
*S100A9*	Forward primer:	ACCCAGACACCCTGAACC
	Reverse primer:	AGCATGATGAACTCCTCGA
*iNOS*	Forward primer:	CAGCGGGATGACTTTCCAA
	Reverse primer:	AGGCAAGATTTGGACCTGCA
*TNF-α*	Forward primer:	CAGCCTCTTCTCCTTCCTGA
	Reverse primer:	GGAAGACCCCTCCCAGATAGA
*IL-10*	Forward primer:	CAAGACCCAGACATCAAGGCG
	Reverse primer:	GCATTCTTCACCTGCTCCACG
*CD206*	Forward primer:	GTCATATCGGGTTGAGCCACT
	Reverse primer:	AATCATTCCGTTCACCAGAGG
*TLR4*	Forward primer:	AGAATGCTAAGGTTGCCGCT
	Reverse primer:	CTATCACCGTCTGACCGAGC
*E-cadherin*	Forward primer:	CCCGGGACAACGTTTATTAC
	Reverse primer:	GCTGGCTCAAGTCAAAGTCC
*N-cadherin*	Forward primer:	CCTTTCAAACACAGCCACGG
	Reverse primer:	TGTTTGGGTCGGTCTGGATG
*Vimentin*	Forward primer:	CTCTGGCACGTCTTGACCTT
	Reverse primer:	ACCATTCTTCTGCCTCCTGC
*VEGF*	Forward primer:	TGCAGATTATGCGGATCAAACC
	Reverse primer:	TGCATTCACATTTGTTGTGCTGTAG
*TGF-β*	Forward primer:	CCCAGCATCTGCAAAGCTC
	Reverse primer:	GTCAATGTACAGCTGCCGCA
*siS100A9*	Sense	GCUUCGAGGAGUUCAUCAUTT
	Antisense	AUGAUGAACUCCUCGAAGCTT
*siNC*	Sense	UUCUCCGAACGUGUCACGUTT
	Antisense	ACGUGACACGUUCGGAGAATT

siNC, nonspecific siRNA; siS100A9, S100A9-siRNA.

M*φ* or CRC cells were treated with TAK-242 (5 µM) and BAY 11-7082 (10 µM) for 1 h prior to *Fn* stimulation. The supernatant was collected after 48 h in the same manner as before.

M*φ* cells were treated with GST (10 µg/ml) or rS100A9 (10 µg/ml). After 24 h, we changed the media with fresh media and collected the supernatant after incubating for another 48 h in the same manner as before.

ALL mentioned conditioned medium (CM) was finally made up by mixing the supernatant with 10% FBS-supplemented fresh medium in a 1:1 ratio to make 5% FBS-containing CM.

### ELISA

Concentration of S100A9, TNF-*α*, and IL-10 in the above-mentioned Conditioned media (CM) with different treatments was measured with specific ELISA kits (JYM, China) according to the manufacturer’s instructions.

### Cell Proliferation Assay

Cell proliferation was assessed by CCK-8 assay using a Cell Counting Kit (Dojindo, Japan) following the manufacturer’s protocol. Briefly, HCT116 and SW480 cells were seeded in 96-well plates at 3,000 cells/well and cultured for described hours. Then CCK8 solution was added. Finally, the absorbance at 450 nm of each well was measured daily using a microplate reader. Each condition was done in quintuplicate, and the experiment was repeated thrice.

### Cell Migration Assay

Cell migration assay was examined to assess the cell migratory capacity of CRC cells using 24-well Transwell cell culture chambers (pore size, 8 μm). Briefly, CRC cells were seeded at a density of 2 × 10^4^ cells per well in serum-free medium at the upper chamber, and the corresponding CM was added in the lower chamber to establish co-culture systems. After incubating at 37°C for 24 h, the transmembrane cells were dried, fixed with methanol, and stained with commercialized crystal violet staining solution (Beyotime, China) and the migration cell number in five to eight fields of each insert was counted under an inverted microscope at a magnification of ×100. The experiment was repeated thrice.

### RNA Isolation and Quantitative Real-Time PCR

Total RNA extraction from the treated cells or tumor tissues was performed with TRIzol reagents (Invitrogen). cDNA samples were synthesized using random primers from 1 mg total RNA with a Reverse Transcription kit (Takara, Japan). The mRNA levels of *S100A9*, *iNOS*, *TNF-α*, *IL-10*, *CD206*, *E-cadherin*, *N-cadherin*, *Vimentin*, *TGF-β, VEGF*, and *TLR4* were analyzed and normalized to the *GAPDH* with the CFX96 real-time PCR detection system (Bio-Rad, USA) using SYBR Green dye (Biomake, China) according to the manufacturer’s instructions. The primers in this study were synthesized by Genscript, and the primer sequence information is shown in **Table 1**.

### Western Blot

The cells of different treatment groups were collected, and total cellular protein was extracted with RIPA buffer containing phosphatase/protease inhibitor after washing three times with ice-cold PBS. The cell lysates were collected after centrifugation, and the concentration of protein was determined by BCA assay. The extraction of xenograft tumor protein was carried out with reference to the kit instructions (BestBio, China) according to the manufacturer’s instructions. Samples containing equal amount of proteins were separated using SDS–PAGE according to the molecular weight of different proteins and transferred to PVDF membranes. Then the membranes were blocked with 5% non-fat dry milk and incubated with anti-S100A9 (Abcam, UK), anti-CD86 (Bioss, China), anti-CD206 (Proteintech, China), anti-total NF-*κ*B p65 (Cell Signaling Technology, USA), anti-Phospho-NF-*κ*B p65 (p-p65) (Ser536) (Cell Signaling Technology, USA), anti-E-cadherin (Immunoway Biotechnology, USA), anti-N-cadherin (Cell Signaling Technology, USA), anti-PCNA (Wanleibio, China), anti-VEFG (Santa Cruz Biotechnology, USA), anti-TGF-*β* (Abcam, UK) or anti-*β*-actin (Cell Signaling Technology, USA), followed by incubation with secondary antibodies conjugated with horseradish peroxidase. The proteins of interest were detected using the SuperSignal West Pico Chemiluminescent Substrate kit. The results were recorded by Bio-Rad Electrophoresis Documentation (Gel Doc 1000, Bio-Rad, USA) and Quantity One Version 4.5.0.

### Flow Cytometry

To investigate M*φ* differentiation, M1 marker (CD86) and M2 marker (CD206) in different treatment groups were analyzed by flow cytometry. Briefly, the prepared cells were collected and washed with PBS and then stained with PE-mouse anti-human CD86 (BioLegend, USA) or PE-mouse anti-human CD206 (BioLegend, USA) for 30 min at 4°C. Cells were then washed and analyzed using FACSVantage SE flow cytometer (Becton-Dickinson, USA).

### Immunofluorescence

M*φ*s on the climbing piece in 24-well culture plates were treated with or without bacterium infection or treated with relevant CM for 48 h. Then the cells were fixed with 4% paraformaldehyde for 20 min, washed with PBS, and permeabilized with 0.01% Triton X-100 for 10 min. After being washed with PBS, the cells were blocked with 10% goat serum for 30 min at room temperature and incubated with anti-CD86 monoclonal antibodies (Santa Cruz, USA) and anti-CD206 antibodies (Proteintech, China) at 4°C overnight. The next day, the cells were rinsed with PBS for clearing the primary antibody, and then incubated with Alexa Fluor 488-conjugated goat anti-mouse secondary antibody (Beyotime, China) or Cy3-conjugated goat anti-rabbit secondary antibody (Beyotime, China) at room temperature for 1 h in the dark, and then washed with PBS, and counterstained the nucleus with DAPI for 10 min. After washing again with PBS and mounting with antifade polyvinylpyrrolidone mounting medium (Beyotime, China), the fluorescent images were observed using confocal microscope (Lecia, Germany).

### Xenograft

Four-week-old female BALB/c nude mice were purchased from Beijing Huafukang Biotechnology. For the xenograft experiments, mice were randomly divided into four groups (n = 3 in each group). HCT116 cells were mixed with equal proportion of M*φ* treated with control *E. coli*, *Fn*, *Fn* + siNC or *Fn* + siS100A9 for 48 h and suspended in 100 µl PBS for the subsequent use. Then the mixed cells (1 × 10^7^) in 100 µl PBS were injected subcutaneously into the posterior flank of each nude mouse to establish the xenograft model. The length and width of a tumor were detected using a caliper every three days. Tumor volumes were calculated according to the following formula: Volume = (width)^2^ × length/2. The mice were sacrificed after 21 days, and the tumor tissues were removed, weighed, fixed in 4% buffered formaldehyde, embedded in paraffin, and sectioned for further immunohistochemical analysis. All the *in vivo* experiments were approved and conducted in accordance with the guidelines established by the University Animal Care and Use Committee for Laboratory Animal Research in Chongqing Medical University (protocol number 2018-003).

### Microbial Fluorescence *In Situ* Hybridization Analysis

The analysis for detecting invasive *Fn* in CRC tissue used *Fn*-targeted probe, FUS664 (FITC-labeled), 5′-CTT GTA GTT CCG C(C/T) TAC CTC-3′ (26). Paraffin-embedded slides were prepared and hybridized with reference to previously described methods (26). Bacteria were counted in five random fields per slide at a magnification of ×100 by an observer blind to the sample status, and the number of bacteria per field was calculated on average. The average number of *Fn* in each field in the case of <5, between 5 and 20, and >20 visualized FUS664 probes was defined as negative, weak, and positive *Fn* abundance, respectively.

### Immunohistochemical Procedures

Indirect immunohistochemistry (IHC) analysis of formalin-fixed and paraffin-embedded tissue sections was performed. In brief, the sections were deparaffinized, dehydrated, boiled in 0.01 M citrate buffer for 10 min, and then incubated with 0.3% H_2_O_2_ in methanol for 10 min to block endogenous peroxidase activity. The sections were incubated with anti-S100A9 (Abcam, UK), anti-CD68 (Santa Cruz, USA), anti-CD86 (Santa Cruz, USA), anti-CD206 (Proteintech, China), anti-E-cadherin (ImmunoWay Biotechnology, USA), and anti-N-cadherin (Cell Signaling Technology, USA) following incubation with secondary antibody tagged with the peroxidase enzyme for 30 min at room temperature and were visualized with 0.05% DAB till the desired brown reaction product was obtained. All slides were counterstained with hematoxylin and then observed using Nikon E400 Light Microscope, and representative photographs were taken.

For double immunofluorescence staining, the sections were incubated with anti-S100A9 together with anti-CD68 (HUABIO, China), anti-CD86 (Santa Cruz, USA), anti-CD206 (Proteintech, China), or anti-cytokeratin 20 (Proteintech, China) antibodies, followed by incubation with secondary antibodies Alexa fluor 647-conjugated anti-mouse IgG and Alexa fluor 488-conjugated anti-rabbit IgG. These sections were also stained with 10 μg/ml DAPI. The fluorescent images were then observed and analyzed using a multi-laser confocal microscope.

### Statistical Analysis

Data from the two groups were analyzed with the two-tailed Student’s t-test and data from three or more groups using one-way ANOVA followed by Newman–Keuls’ multiple comparison test. All experiments were independently repeated three times. The statistical analyses were carried out using SPSS version 13.0. Statistical differences are presented at probability levels of p <0.05, p <0.01, and p <0.001.

## Results

### Involvement of *Fn* in M2-Like Polarization of M*φ*

**Figure 1:**
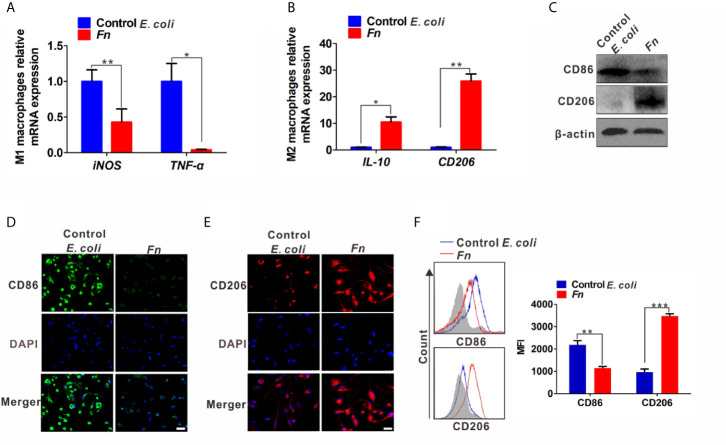
*Fn* promotes M2-like polarization of M*φ in vitro*. **(A, B)** qPCR analysis for mRNA levels of M1 markers (iNOS and TNF-α) and M2 markers (IL-10 and CD206) in M*φ* co-cultured with control *E. coli i* or *Fn* for 24 h. **(C)** Western blot analysis of M1 marker (CD86) and M2 marker (CD206) expression in M*φ* co-cultured with control *E. coli* or *Fn* for 48 h. **(D, E)** Representative immunofluorescence images of CD86^+^ and CD206^+^ M*φ* after treatment with control *E. coli* or *Fn* for 48 h. CD86 was stained with Alexa Fluor 488 (green), CD206 was stained with Cy3 (red). Scale bars: 50 µm. **(F)** Flow cytometry analysis was performed to detect CD86^+^ and CD206^+^ M*φ* after treatment with control *E. coli* or *Fn* for 48 h. A statistical mean fluorescence intensity (MFI) for CD86^+^ and CD206^+^ M*φ* is shown in the right panel. Data were expressed as means ± SD in three independent experiments. *p < 0.05, **p < 0.01, ***p < 0.001.

To investigate whether the phenotypic differentiation of Mφ is affected by *Fn* infection *in vitro*, M1 markers (inducible nitric oxide synthase, iNOS; tumor necrosis factor, TNF-α; the B7-related cell surface proteins B7-2, CD86) and M2 markers (interleukin-10, IL-10; the mannose receptor, MRC/CD206) were determined. Compared to the control *E. coli*-challenged M*φ*, *Fn*-challenged M*φ* harbored significantly lower mRNA level of M1 markers *iNOS* and *TNF-α* but significantly higher mRNA level of M2 markers *IL-10* and *CD206* (**Figures 1A, B**). Similar tendency for protein levels of TNF-α and IL-10 in the supernatants was confirmed by ELISA (**Figure S1**). These results suggested that *Fn* may be involved in M2-like polarization. Marked CD206 expression but less CD86 expression was detected in *Fn*-challenged M*φ* by western blot (**Figure 1C**). High intensity of the signal for CD206 but low intensity of signal for CD86 was also confirmed by immunofluorescence and flow cytometry analyses (**Figures 1D–F**). Collectively, these findings indicated that *Fn* might contribute to M2-like M*φ* differentiation.

### Elevated S100A9 Expression in *Fn*-Challenged M*φ* and CRC Cells

**Figure 2:**
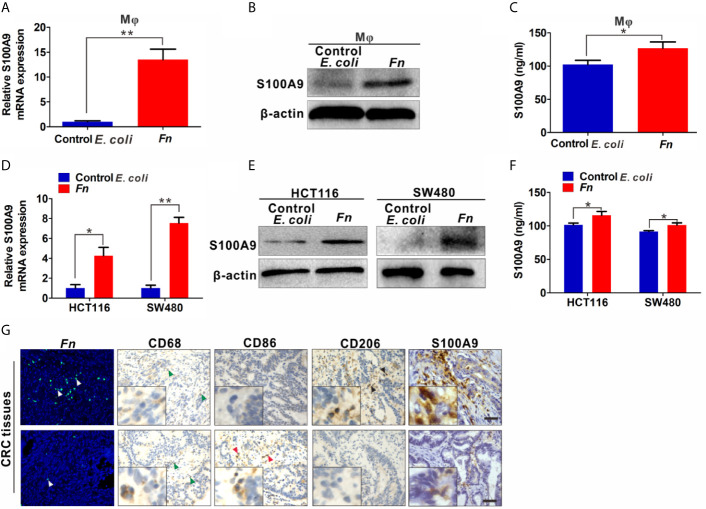
S100A9 expression is up-regulated in *Fn*-challenged M*φ* and CRC cells. **(A–F)** M*φ* and CRC cells were co-cultured with control *E. coli* or *Fn*. S100A9 levels were detected by qPCR **(A, D)**, Western blot **(B, E)** and ELISA **(C, F)**. **(G)** Representative images of CD68^+^ M*φ*, CD86^+^ M*φ*, CD206^+^ M*φ*, and S100A9 expression in the TME of *Fn*-positive CRC tissues. White arrow: *Fn*; blue arrow: CD68^+^ M*φ*; red arrow: CD86^+^ M*φ*; black arrow: CD206^+^ M*φ*. Scale bars: 50 µm. Data were expressed as means ± SD in three independent experiments. *p < 0.05, **p < 0.01.

Increased level of S100A9 is related to the infection of multiple pathogens and participates in disease progression by regulating inflammatory immunity (27, 28). Therefore, we analyzed the S100A9 level in *Fn*-challenged M*φ* and CRC cells. The mRNA level of *S100A9* was significantly up-regulated in the *Fn*-challenged M*φ* (**Figure 2A**). Consistently, its protein level in *Fn*-challenged M*φ* as well as the supernatant was also markedly increased compared to the control groups (**Figures 2B, C**). In line with the finding, *Fn-*infected CRC cells as well as the supernatant also showed high level of S100A9 than the control group (**Figures 2D–F**). These data suggested that S100A9 may be elevated in the microenvironment of CRC with *Fn* infection. Next, we evaluated the enrichment of *Fn* and detected CD68^+^ M*φ*, CD86^+^ M1-like M*φ*, CD206^+^ M2-like M*φ*, and S100A9 expression in CRC tissues. As expected, there was more M*φ* infiltration in *Fn*-positive CRC tissues compared to *Fn*-negative CRC tissues, and most of them were M2-like M*φ*s accompanied by higher levels of S100A9 in the TME (**Figures 2G** and **S2**). The results suggested that S100A9 expression is elevated in *Fn*-infected M*φ* and CRC cells, which may be associated with M2-like M*φ* polarization in CRC tissues.

### S100A9 Is Involved in M2-Like M*φ* Polarization in the Inflammatory Microenvironment of CRC With *Fn* Infection

**Figure 3:**
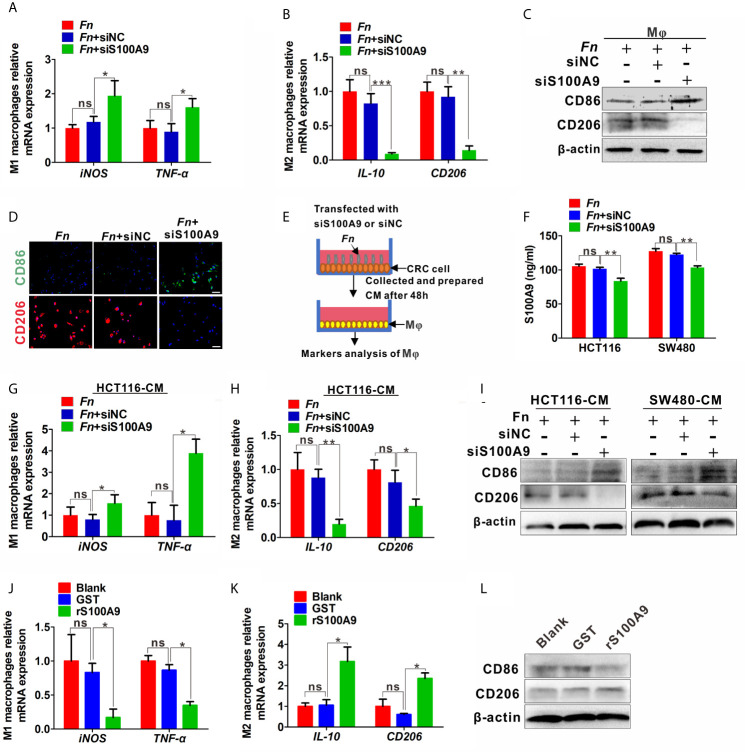
S100A9 is involved in M2-like M*φ* polarization in the presence of *Fn* in CRC. **(A–C)** M*φ*s were transfected with siNC or siS100A9 and subsequently co-cultured with *Fn*. The levels of M1 markers and M2 markers in M*φ* were detected by qPCR **(A, B)** and Western blot **(C)**. **(D)** Representative immunofluorescence images of CD86^+^ and CD206^+^ in M*φ* infected with *Fn* and then transfected with siNC or siS100A9. CD86 was stained with Alexa Fluor 488 (green); CD206 was stained with Cy3 (red). Scale bars: 50 µm. **(E, F)** HCT116 and SW480 cells were transfected with siNC or siS100A9 and then infected with *Fn*. After 48 h, the supernatant of each group was collected and used to culture M*φ* in the next experiment **(E)**. ELISA analysis for S100A9 expression in the supernatant of HCT116 and SW480 cells **(F)**. **(G–I**) M*φ*s were co-cultured with the CM from CRC cells. The levels of M1 markers and M2 markers in M*φ* were detected by qPCR **(G, H)** and Western blot **(I)**. **(J–L)** M*φ*s were treated with GST or rS100A9. The levels of M1 markers and M2 markers in M*φ* were detected by qPCR **(J, K)** and Western blot **(L)**. Data were expressed as means ± SD in three independent experiments. ns, not significant. *p < 0.05; **p < 0.01; ***p < 0.001.

The results above showed that S100A9 was highly expressed in *Fn*-infected CRC, but the direct evidence about the relation of higher S100A9 and M2-like M*φ* polarization needs to be further studied. Therefore, we analyzed the M*φ* polarization status in siS100A9-transfected M*φ* cultured with *Fn*. As expected, S100A9 expression was successfully reduced in the supernatant of siS100A9-transfected M*φ* with *Fn* infection (**Figure S3**). Knockdown of S100A9 expression obviously blocked *Fn*-induced increase of mRNA levels of M2 markers *IL-10* and *CD206* but improved *Fn*-induced decrease of mRNA levels of M1 marker *iNOS* and *TNF-α* (**Figures 3A, B**). The protein levels of TNF-α and IL-10 in the supernatants were confirmed by ELISA (**Figure S4**). Consistently, down-regulated M2 marker CD206 and up-regulated M1 marker CD86 were observed in siS100A9-transfected M*φ* infection with *Fn* by western blot (**Figure 3C**). Low intensity of signal for CD206 but high signal for CD86 was also confirmed in siS100A9-transfected M*φ* infection with *Fn* by immunofluorescence assay (**Figure 3D**). To better illustrate the effect of S100A9 on M2-like M*φ* polarization in the *Fn*-infected CRC inflammation microenvironment, CRC cells were transfected with siS100A9 followed by *Fn* infection, and then CM was collected for further culturing with M*φ* (**Figure 3E**). siS100A9 transfection significantly inhibited the secretion of S100A9 protein in *Fn*-infected CRC cells compared to the control groups (**Figure 3F**). Moreover, M*φ* tended to be polarized towards M1-like M*φ* (mRNA levels: *iNOS* ^high^, *TNF-α* ^high^, *IL-10* ^low^, *CD206* ^low^; protein levels: CD86^high^, CD206^low^) after culturing with the CM from CRC cells transfected with siS100A9 followed by *Fn* infection (**Figures 3G–I**). In contrast, after treatment with rS100A9 at a final concentration of 10 μM for 48 h, M*φ*s were apt to be M2 activated (mRNA levels: *iNOS* ^low^, *TNF-α* ^low^; *IL-10* ^high^, *CD206* ^high^; protein levels: CD86^low^, CD206^high^) (**Figures 3J–L**). In conclusion, these results directly supported that elevated S100A9 favors the M2 polarization of M*φ* in the TME of *Fn*-infected CRC.

### *Fn*-Challenged M2-like M*φ*, Which Is Mediated by S100A9, Promotes the Proliferation and Migration of CRC Cells

**Figure 4:**
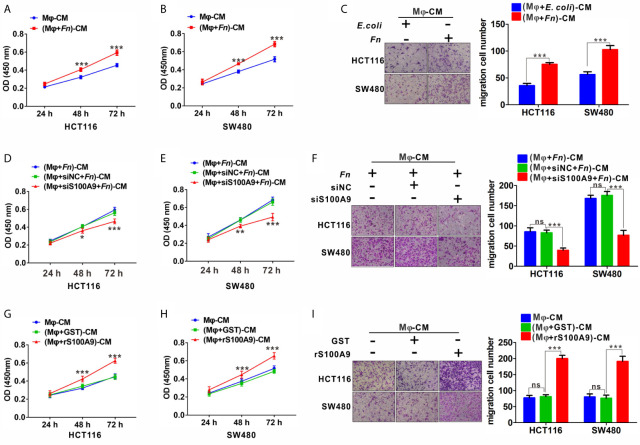
*Fn*-challenged M2-M*φ*, which was mediated by S100A9, promotes the proliferation and migration of CRC cells. **(A, B)** CCK8 assay was used to determine the proliferation ability of CRC cells co-cultured with (M*φ* + *E. coli*)-CM or (M*φ* + *Fn*)-CM for 24, 48, and 72 h. **(C)** Transwell assay was used to determine the migration ability of CRC cells co-cultured with (M*φ* + *E*. *coli*)-CM or (M*φ* + *Fn*)-CM for 24 h. Magnification, 100×. **(D, E)** CCK8 assay was used to determine the proliferation ability of CRC cells co-cultured with (M*φ* + *Fn*)-CM, (M*φ* + siNC + *Fn*)-CM, (M*φ* + siS100A9 + *Fn*)-CM for 24, 48, and 72 h. **(F)** Transwell assay was used to determine the migration ability of CRC cells co-cultured with (M*φ* + *Fn*)-CM, (M*φ* + siNC + *Fn*)-CM, (M*φ* + siS100A9 + *Fn*)-CM for 24 h. Magnification, 100×. **(G, H)** CCK8 assay was used to determine the proliferation ability of CRC cells co-cultured with untreated M*φ*-CM, (M*φ* + GST)-CM and (M*φ* + rS100A9)-CM for 24, 48, and 72 h. **(I)** Transwell assay was used to determine the migration ability of CRC cells co-cultured with untreated M*φ*-CM, (M*φ* + GST)-CM and (M*φ* + rS100A9)-CM for 24 h. Magnification, 100×. In **(A, B, D, E, G, H)**, data shown are mean absorbances ± SD. In **(C, F, I)**, data shown are mean migrating cells ± SD. ns, not significant. *p < 0.05, **p < 0.01, ***p < 0.001.

To further investigate the effects of *Fn-*challenged M*φ* on the proliferation and migration ability of HCT116 and SW480 cells, CCK8 and Transwell assays were conducted after CRC cells were treated with various Mφ CM. There was a significant increase in the proliferation (**Figures 4A, B**) and migration (**Figure 4C**) abilities of HCT116 and SW480 cells cultured with *Fn*-challenged M*φ* CM [(M*φ* + *Fn*)-CM], while the effect would be reversed after silencing S100A9 expression in *Fn*-challenged Mφ CM [(M*φ* + siS100A9 + *Fn*)-CM)] (**Figures 4D–F**). The remarkably enhanced ability of proliferation (**Figures 4G, H**) and migration (**Figure 4I**) of HCT116 and SW480 cells was confirmed again when these cells were cultured with rS100A9-treated M*φ* CM [(M*φ* + rS100A9)-CM). In addition, epithelial-to-mesenchymal transition (EMT)-related genes *N-cadherin* and *Vimentin* were up-regulated, but *E-cadherin* was decreased in HCT116 cells cultured with (M*φ* + *Fn*)-CM (**Figure S5**). These results, along with those aforementioned, suggested that *Fn*-challenged M2-like M*φ*, which was mediated by S100A9 in the TME, confers onto CRC cells a more malignant phenotype.

### *Fn* Up-Regulates S100A9 Expression and Promotes M2-Like Polarization *via* the TLR4/NF-*κ*B Pathway

**Figure 5:**
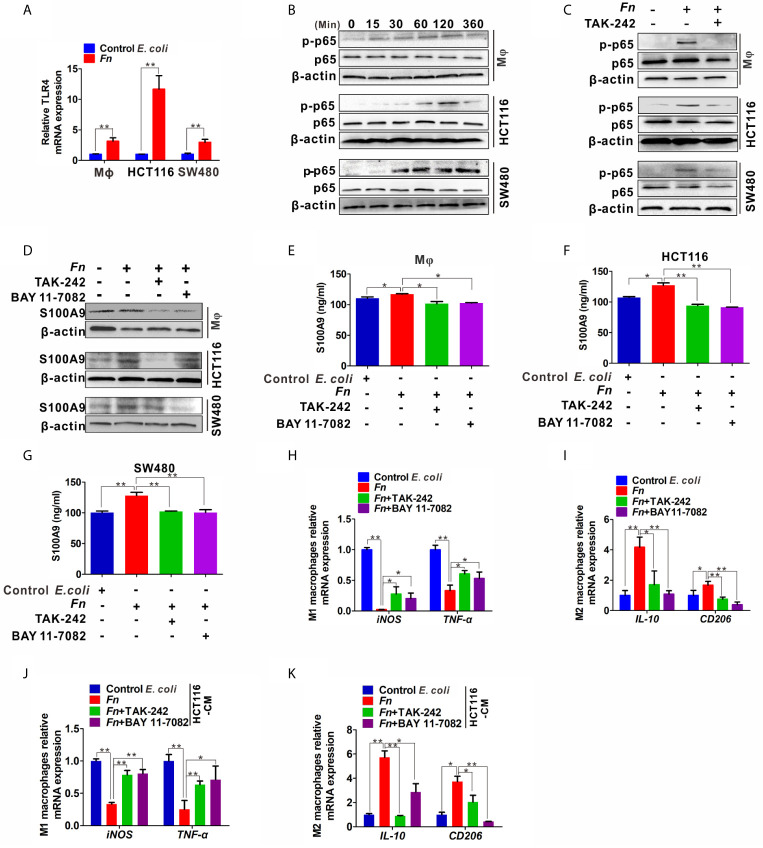
Expression of S100A9 and M2-like M*φ* polarization is regulated by TLR4/NF-*κ*B activation caused by *Fn*. **(A)** qPCR analysis of TLR4 expression in M*φ* and CRC cells co-cultured with control *E. coli* or *Fn* for 48 h. **(B)** Western blot analysis of p65 and p-p65 expression in M*φ* and CRC cells co-cultured with *Fn* for 0,15, 30, 60, 120 and 360 min. **(C)** Western blot analysis of p-p65 level in M*φ* and CRC cells pretreated with or without inhibitor TAK-242 for 60 min and then co-cultured with control *E. coli* or *Fn* for 120 min. **(D)** Western blot analysis of S100A9 level in M*φ* and CRC cells pretreated with or without inhibitors TAK-242 and NF-*κ*B for 60 min and then co-cultured with control *E. coli* or *Fn* for 48 h. **(E–G)** ELISA analysis of S100A9 level in the CM of M*φ* and CRC cells pretreated with or without inhibitors TAK-242 and Bay 11-7082 for 60 min and then co-cultured with control *E. coli* or *Fn* for 48 h. **(H–K)** qPCR analysis for mRNA levels of M1 markers (iNOS and TNF-α) and M2 markers (IL-10 and CD206) in M*φ*. The cells were pretreated with or without inhibitors TAK-242 and Bay 11-7082 for 60 min and then co-cultured with control *E. coli* or *Fn* for 24 h **(H, I)** or co-cultured with the CM of HCT116 cells for 24 h **(J, K)**. The CM of HCT116 cells were pretreated with or without inhibitors TAK-242 and Bay 11-7082 for 60 min and then co-cultured with control *E. coli* or *Fn* for 48 h. Data were expressed as means ± SD in three independent experiments. *p < 0.05, **p < 0.01.

To further examine the mechanisms by which *Fn* engages in S100A9 expression and M2 polarization, we focused on TLR4/NF-*κ*B pathway, which is substantially activated in CRC with *Fn* enrichment (21) and determine whether it takes part in the effect. The mRNA levels of TLR4 in M*φ*, HCT116, and SW480 cells were obviously increased in response to *Fn* infection (**Figure 5A**). Phospho-NF-*κ*B p65 (p-p65) levels in M*φ*, HCT116, and SW480 cells were also increased with time and reached a peak almost at 120 min, while there was no obvious change in the total p65 (**Figure 5B**). *Fn*-induced p-p65 in the three cell lines was inhibited after TLR4 inhibitor TAK-242 stimulation (**Figure 5C**), suggesting TLR4 mediated *Fn*-mediated NF-*κ*B activation. Especially, treatment with TLR4 inhibitor TAK-242 and NF-*κ*B inhibitor BAY 11-7082 also inhibited S100A9 protein levels in *Fn*-challenged M*φ* and CRC cells (**Figure 5D**) as well as its levels in cell supernatant (**Figures 5E–G**). Exposure of *Fn*-challenged M*φ* to the TAK-242 or BAY 11-7082 failed to induce M2-like M*φ* (mRNA levels: *iNOS* ^high^, *TNF-α* ^high^, *IL-10* ^low^, *CD206* ^low^) (**Figures 5H, I**). Consistently, this change in M*φ* phenotype was again proved when cultured with the CM from *Fn*-challenged CRC cells, which were pretreated with TAK-242 and BAY 11-7082 (**Figures 5J, K**). Altogether, these results indicated that the TLR4-mediated NF-*κ*B pathway is partially responsible for M2-like polarization induced by S100A9 in response to *Fn* infection in CRC.

### Accelerated Growth of the Subcutaneous Tumor by M2-Like M*φ* Induced by *Fn*

**Figure 6:**
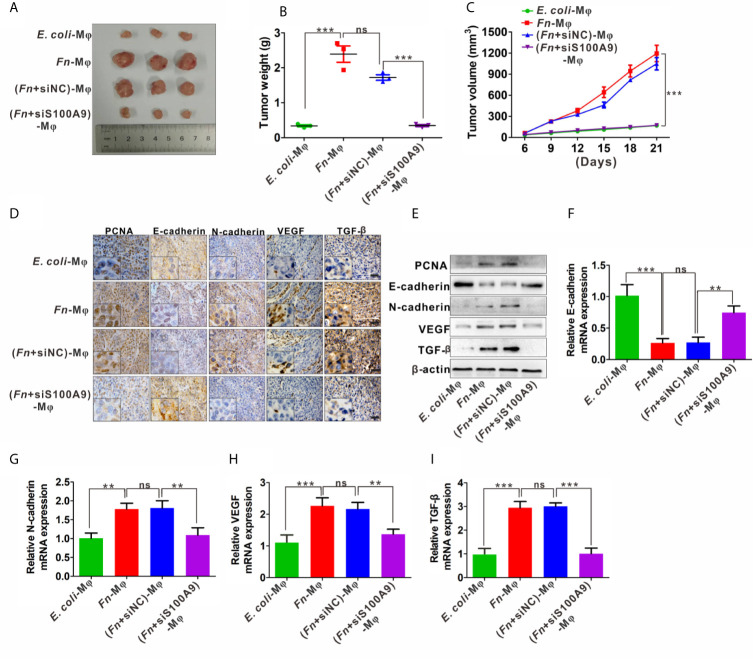
*Fn*-caused M2 polarization mediated by S100A9 promotes the growth of subcutaneous tumor. **(A)** Representative images of tumors in mice co-injected with equal amount of HCT116 cells and control *E. coli* -treated, *Fn*-treated, (*Fn* + siNC)-treated or (*Fn* + siS100A9)-treated M*φ*. **(B)** Images of tumor weights in different groups, n = 3/group. **(C)** Statistical analysis of tumor volumes in different groups, n = 3/group. **(D)** Representative immunohistochemistry images of PCNA, E-cadherin, N-cadherin, VEGF, and TFG-*β* proteins in representative xenograft tumor sections. Scale bars: 50 µm. **(E)** Western blot analysis of PCNA, E-cadherin, N-cadherin, VEGF, and TFG-*β* protein levels in representative xenograft tumor. **(F–I)** qPCR analysis of E-cadherin, N-cadherin, VEGF, and TGF-*β* expression in each xenograft tumor, n = 3/group. Data were expressed as means ± SD in three independent experiments. ns, not significant. **p < 0.01, ***p < 0.001.

In the xenograft nude mouse models, HCT116 and differently treated M*φ* were inoculated into nude mice. The results from the nude mouse xenograft model showed that co-injection with HCT116/*Fn*-treated M*φ* significantly accelerated the tumor growth, and this effect was partially blocked by silencing S100A9 expression in M*φ* in the nude mice co-injected with HCT116/(*Fn* + siS1A00A9)-treated M*φ* (**Figure 6A**). Tumor growth curve and the average weight of each group also showed the same tendency (**Figures 6B, C**). In addition, IHC results showed that the protein levels of proliferation-related marker PCNA, EMT-related marker E-cadherin and N-cadherin, and tumor-promoting molecules VEGF and TGF-*β* were higher in tumors of nude mice co-injected with HCT116/*Fn*-treated M*φ* than those of the compared control groups, while these protein levels were decreased in the nude mice co-injected with HCT116/(*Fn* + siS1A00A9)-treated M*φ* (**Figure 6D**). Western blot and qPCR results about the levels of PCNA, E-cadherin, N-cadherin, VEGF, TGF-*β* in each xenograft tumor were also consistent with the result of IHC (**Figures 6E–I**). These data suggested that the interaction between HCT116 and *Fn*-treated M*φ* promotes tumor growth in nude mice *in vivo*, which may be involved in *Fn*-induced M2-like polarization mediated by S100A9.

## Discussion

**Figure 7:**
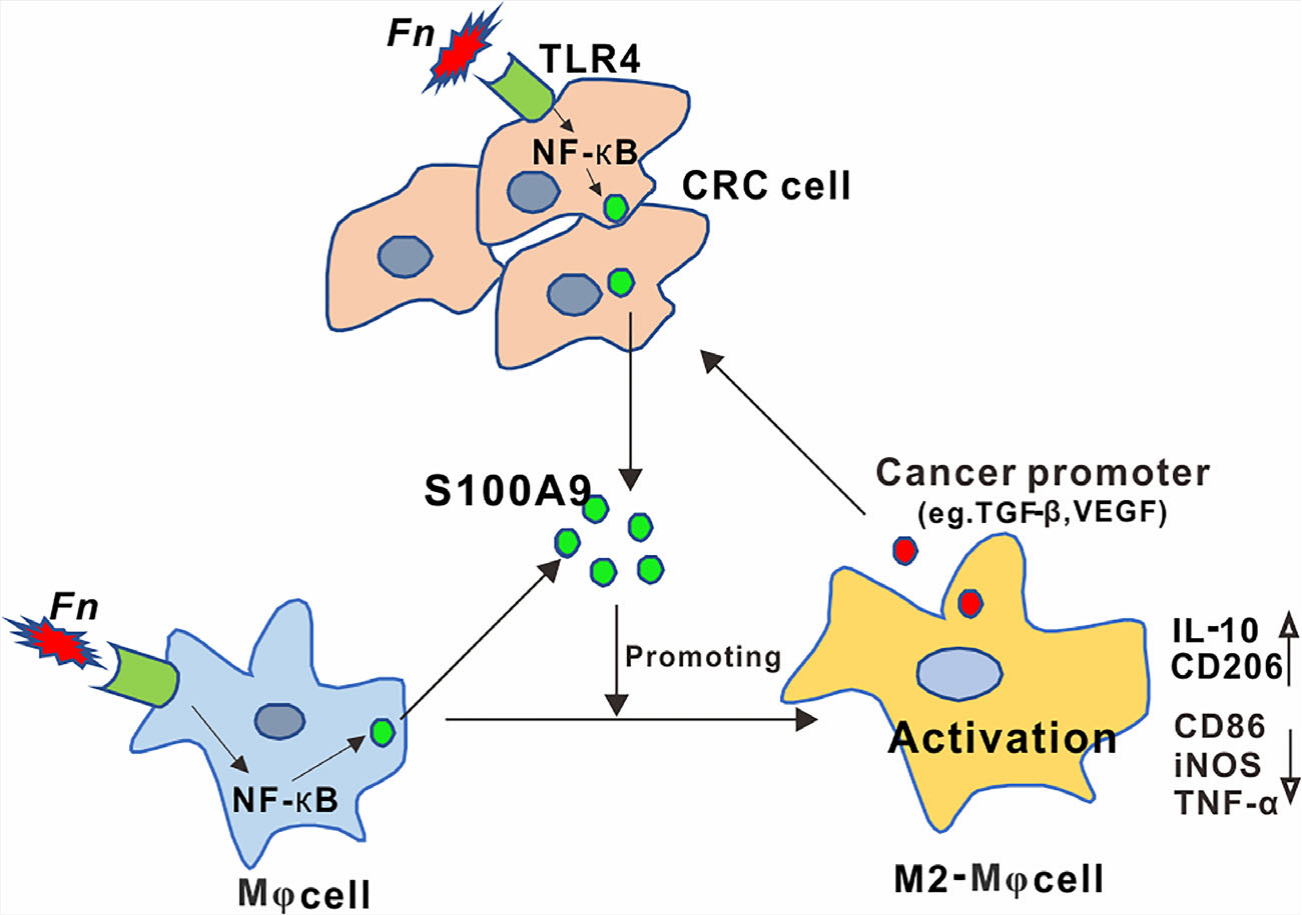
Schematic diagram of the relationship among *Fn*, M2 polarization, and CRC progression. Activation of the TLR4/NF-*κ*B cascade in M*φ* and CRC cells by *Fusobacterium nucleatum* infection mediated high levels of S100A9 in CRC microenvironment, which led to M2-like M*φ* phenotype showing increased IL-10 and CD206 levels, contributing to CRC malignance.

Identifying specific carcinogenic microorganisms remains the focus of CRC research (29). Recently, among these identified and published CRC-related gut microbes, *Fn* has attracted the most attention. Although involvement of *Fn* in immunosuppressive TME has been indicated, the detailed mechanisms by which *Fn* participated in regulating the activation of immune cells have not been well-elucidated. Here, we demonstrated a regulatory role of *Fn* in CRC TME on M2-like polarization, which is mediated by activation of TLR4-dependent NF-*κ*B/S100A9 signaling pathway in M*φ*s and CRC cells (**Figure 7**).

M*φ*s are dynamic and heterogeneous cells whose differentiation, tissue distribution, and responsiveness to stimuli are governed by different mechanisms (30). Based on their response to different microenvironmental signaling factors, M*φ*s can be differentiated into two classifications, namely, M1-like M*φ* sand M2-like M*φ*s. M1-like Mφ mainly plays a pro-inflammatory and anti-tumor role, while M2-like Mφ is often considered to be tumor-promoting (31). Viral, parasitic, fungal, and bacterial pathogens can induce M2 activation in M*φ* and further promote pathogen-induced inflammatory injury and even canceration (32). Numerous studies have elucidated *Fn*, a Gram-negative oral commensal anaerobe, which is one of the most prevalent species in oral inflammation diseases and extraoral infections (33, 34). At the same time, *Fn* has been reported to be highly enriched in CRC tissues and helps tumor cells to achieve immune evasion by inhibiting immune cell activity in TME (13, 35–37). However, the presence and activation state of M*φ* in *Fn-*infected CRC remain to be elucidated. In this study, we determined that *Fn*-challenged M*φ*s were inclined to M2-like activation, which in turn enhances the proliferation and migration of CRC cells *in vitro*. Furthermore, our results from the xenograft nude mouse also supported a positive association between *Fn*-challenged M2-like M*φ* and tumor growth. Altogether, our results suggest that *Fn*-induced M2-like polarization facilitated CRC progression.

Compelling links are beginning to appear among bacteria, immune escape, and CRC. Base on *Fn* abundance in CRC along with weaken host anti-tumor immunity, there is a great need for further mechanistic understanding of CRC to find a novel target molecule. S100A9, originally known as immunogenic protein, is mainly expressed by bone marrow-derived cells such as mononuclear, M*φ*, and neutrophils (17). Besides, S100A9 induces positive feedback and promotes further leukocyte recruitment under pathological conditions associated with inflammatory infection (38). Experimental data indicated that S100A9 presents abundant expression in various inflammation-associated human cancers, no doubt including CRC (17, 18). Our previous work confirmed elevated S100A9 levels in CRC, stimulating survival and migration of CRC cells (18). Here, we further determined that high S100A9 levels in CRC may be associated with *Fn* infection. Moreover, the number of tumor-infiltrating M2-like M*φ* was more in *Fn*-positive CRC tissues accompanied by higher S100A9 expression than that in *Fn*-negative tissues with lower S100A9 expression. These results further prompt us to infer that S100A9 may play a potential role in regulating M*φ* toward M2-like phenotype. Previously, tumor-infiltrating S100A9-positive inflammatory or immune cell tissues were closely related to the pathological stage of CRC (22). S100A9 was also reported to be involved in THP-1 cells differentiated into MDSCs or M2-like M*φ* stimulated by myofibroblast-based CM (39). Here, our results demonstrated that up-regulation of S100A9 expression from *Fn*-infected M*φ* and CRC cells in the TME was partially responsible for M2-like M*φ* activation state. Not surprisingly, the proliferation and migration ability of CRC cells co-cultured with *Fn*-treated M*φ* would be weakened by silencing S100A9 expression in the TME of CRC.

In this study, we attempted to uncover the mechanism for regulation of S100A9 expression and M2-like M*φ* polarization by *Fn* infection. As an inflammation/immunity mediator, high levels of S100A9 have been indicated and aggravated mutiple pathogen infections, including coxsackievirus B3 (CVB3)-induced myocarditis (27), streptococcus pneumoniae (SP)-related otitis media (OM) (40), and influenza A virus-related pneumonia (28). The TLR4/NF-*κ*B signal is often activated in pathogen infection. Influenza A virus could up-regulate S100A9 expression and synergistically activate TLR4 signaling cascade in lung M*φ* and endothelial cells, exaggerating pro-inflammatory response, cell-death, and virus pathogenesis (28). Our previous study also found HBV-induced NF-*κ*B activation enhances transcription of S100A9 by binding to its promoter, contributing to HCC malignancy (16). In human cytomegalovirus (HCMV)-related inflammatory breast cancer, NF-*κ*B activation could result in M2-like M*φ* polarization accompanied with the secretion of tumor-promoting M2-like cytokines (41, 42). Thus, we speculated that *Fn* infection may facilitate CRC malignancy involving M*φ* activation, either directly or indirectly, *via* activating specific signal pathways such as that of TLR4-dependent NF-*κ*B. Now we have shown that PAMP extracted from *Fn* and *Fn* itself could be recognized by TLR4, which further activates its downstream signaling cascade in CRC (8, 21). Also, NF-*κ*B, as a widely acknowledged downstream effector of TLR4 signaling, is highly activated in *Fn*-associated CRC (6). Interestingly, we found a significant increase in the expression of TLR4 and time-dependent activation of NF-*κ*B caused by *Fn* infection in M*φ* and CRC cells, which was inhibited by TLR4 inhibitor TAK-242. In addition, S100A9 expression and M2 activation were also significantly suppressed when the TLR4/NF-*κ*B pathway was blocked. All the evidence above suggested that interference with TLR4/NF-*κ*B/S100A9 cascade should be given enough attention in the treatment of *Fn*-associated CRC.

In conclusion, the current findings demonstrate that S100A9 plays a critical role in regulating M2-like M*φ* polarization in *Fn*-infected CRC microenvironment, and targeting TLR4/NF-*κ*B/S100A9 cascade may attenuate the immunosuppressive effect and serve as promising immunotherapy strategy for *Fn*-associated CRC.

## Data Availability Statement

The raw data supporting the conclusions of this article will be made available by the authors without undue reservation.

## Ethics Statement

The studies involving human participants were reviewed and approved by the Ethics Committee of Chongqing Medical University. The patients/participants provided their written informed consent to participate in this study. The animal study was reviewed and approved by the Ethics Committee of Chongqing Medical University.

## Author Contributions

LH and YL performed the experiments, analyzed the data, and wrote the manuscript. XK, RW, QP, and YZ analyzed the data. LD and LZ conceived ideas, oversaw the research, and co-wrote the manuscript. All authors contributed to the article and approved the submitted version.

## Funding

This work was supported by The National Natural Science Foundation of China (Grant No. 82072364 to LD), Natural Science Foundation of Chongqing (Grant No. cstc2019jcyj-msxmX0864 to LD), and Chongqing Health Commission (Grant No. 2020FYYX038 to LD).

## Conflict of Interest

The authors declare that the research was conducted in the absence of any commercial or financial relationships that could be construed as a potential conflict of interest.

## References

[1] Song M Garrett WS Chan AT. Nutrients, Foods, and Colorectal Cancer Prevention. Gastroenterology (2015) 148(6):1244–60.e1216. 10.1053/j.gastro.2014.12.035 25575572 PMC4409470

[2] Eser S Chang J Charalambous H Silverman B Demetriou A Yakut C. Incidence Patterns of Colorectal Cancers in Four Countries of the Middle East Cancer Consortium (Cyprus, Jordan, Israel, and Izmir, Turkey) Compared With Those in the United States Surveillance, Epidemiology, and End Results Program. Turk J Gastroenterol (2018) 29(1):36–44. 10.5152/tjg.2018.17263 29391306 PMC6322602

[3] Siegel RL Miller KD Goding Sauer A Fedewa SA Butterly LF Anderson JC. Colorectal Cancer Statistics, 2020. CA Cancer J Clin (2020) 70(3):145–64. 10.3322/caac.21601 32133645

[4] Sun J Kato I. Gut Microbiota, Inflammation and Colorectal Cancer. Genes Dis (2016) 3(2):130–43. 10.1016/j.gendis.2016.03.004 PMC5221561 28078319

[5] Garcia-Castillo V Sanhueza E McNerney E Onate SA Garcia A. Microbiota Dysbiosis: A New Piece in the Understanding of the Carcinogenesis Puzzle. J Med Microbiol (2016) 65(12):1347–62. 10.1099/jmm.0.000371 27902422

[6] Zhang S Cai S Ma Y. Association Between Fusobacterium Nucleatum and Colorectal Cancer: Progress and Future Directions. J Cancer (2018) 9(9):1652–9. 10.7150/jca.24048 PMC5950595 29760804

[7] Strauss J Kaplan GG Beck PL Rioux K Panaccione R Devinney R. Invasive Potential of Gut Mucosa-Derived Fusobacterium Nucleatum Positively Correlates With IBD Status of the Host. Inflammation Bowel Dis (2011) 17(9):1971–8. 10.1002/ibd.21606 21830275

[8] Chen T Li Q Wu J Wu Y Peng W Li H. Fusobacterium Nucleatum Promotes M2 Polarization of Macrophages in the Microenvironment of Colorectal Tumours Via a TLR4-Dependent Mechanism. Cancer Immunol Immunother (2018) 67(10):1635–46. 10.1007/s00262-018-2233-x PMC11028377 30121899

[9] Mima K Nishihara R Qian ZR Cao Y Sukawa Y Nowak JA. Fusobacterium Nucleatum in Colorectal Carcinoma Tissue and Patient Prognosis. Gut (2016) 65(12):1973–80. 10.1136/gutjnl-2015-310101 PMC4769120 26311717

[10] Flanagan L Schmid J Ebert M Soucek P Kunicka T Liska V. Fusobacterium Nucleatum Associates With Stages of Colorectal Neoplasia Development, Colorectal Cancer and Disease Outcome. Eur J Clin Microbiol Infect Dis (2014) 33(8):1381–90. 10.1007/s10096-014-2081-3 24599709

[11] Kaplan CW Ma X Paranjpe A Jewett A Lux R Kinder-Haake S. Fusobacterium Nucleatum Outer Membrane Proteins Fap2 and RadD Induce Cell Death in Human Lymphocytes. Infect Immun (2010) 78(11):4773–8. 10.1128/IAI.00567-10 PMC2976331 20823215

[12] Chaushu S Wilensky A Gur C Shapira L Elboim M Halftek G. Direct Recognition of Fusobacterium Nucleatum by the NK Cell Natural Cytotoxicity Receptor NKp46 Aggravates Periodontal Disease. PLoS Pathog (2012) 8(3):e1002601. 10.1371/journal.ppat.1002601 22457623 PMC3310798

[13] Gur C Ibrahim Y Isaacson B Yamin R Abed J Gamliel M. Binding of the Fap2 Protein of Fusobacterium Nucleatum to Human Inhibitory Receptor TIGIT Protects Tumors From Immune Cell Attack. Immunity (2015) 42(2):344–55. 10.1016/j.immuni.2015.01.010 PMC4361732 25680274

[14] Kwon CH Moon HJ Park HJ Choi JH Park DY. S100A8 and S100A9 Promotes Invasion and Migration Through p38 Mitogen-Activated Protein Kinase-Dependent NF-kappaB Activation in Gastric Cancer Cells. Mol Cells (2013) 35(3):226–34. 10.1007/s10059-013-2269-x PMC3887919 23456298

[15] Srikrishna G. S100A8 and S100A9: New Insights Into Their Roles in Malignancy. J Innate Immun (2012) 4(1):31–40. 10.1159/000330095 21912088 PMC3250655

[16] Duan L Wu R Zhang X Wang D You Y Zhang Y. Hbx-Induced S100A9 in NF-kappaB Dependent Manner Promotes Growth and Metastasis of Hepatocellular Carcinoma Cells. Cell Death Dis (2018) 9(6):629. 10.1038/s41419-018-0512-2 29795379 PMC5967311

[17] Shabani F Farasat A Mahdavi M Gheibi N. Calprotectin (S100A8/S100A9): A Key Protein Between Inflammation and Cancer. Inflammation Res (2018) 67(10):801–12. 10.1007/s00011-018-1173-4 30083975

[18] Duan L Wu R Ye L Wang H Yang X Zhang Y. S100A8 and S100A9 are Associated With Colorectal Carcinoma Progression and Contribute to Colorectal Carcinoma Cell Survival and Migration Via Wnt/beta-catenin Pathway. PLoS One (2013) 8(4):e62092. 10.1371/journal.pone.0062092 23637971 PMC3637369

[19] Huang M Wu R Chen L Peng Q Li S Zhang Y. S100a9 Regulates Mdscs-Mediated Immune Suppression Via the RAGE and TLR4 Signaling Pathways in Colorectal Carcinoma. Front Immunol (2019) 10:2243. 10.3389/fimmu.2019.02243 31620141 PMC6759487

[20] Podgorska M Oldak M Marthaler A Fingerle A Walch-Ruckheim B Lohse S. Chronic Inflammatory Microenvironment in Epidermodysplasia Verruciformis Skin Lesions: Role of the Synergism Between HPV8 E2 and C/EBPbeta to Induce Pro-Inflammatory S100A8/A9 Proteins. Front Microbiol (2018) 9:392. 10.3389/fmicb.2018.00392 29563902 PMC5845987

[21] Yang Y Weng W Peng J Hong L Yang L Toiyama Y. Fusobacterium Nucleatum Increases Proliferation of Colorectal Cancer Cells and Tumor Development in Mice by Activating Toll-Like Receptor 4 Signaling to Nuclear Factor-kappaB, and Up-Regulating Expression of Microrna-21. Gastroenterology (2017) 152(4):851–866 e824. 10.1053/j.gastro.2016.11.018 27876571 PMC5555435

[22] Bassorgun CI Unal B Erin N Ozluk A Uzun OC Elpek GO. S100A8 and S100A9 Positive Cells in Colorectal Carcinoma: Clinicopathological Analysis. Gastroenterol Res Pract (2014) 2014:943175. 10.1155/2014/943175 25371673 PMC4211213

[23] Nemeth J Stein I Haag D Riehl A Longerich T Horwitz E. S100A8 and S100A9 are Novel Nuclear Factor Kappa B Target Genes During Malignant Progression of Murine and Human Liver Carcinogenesis. Hepatology (2009) 50(4):1251–62. 10.1002/hep.23099 19670424

[24] Wang Q Zhao L Xu C Zhou J Wu Y. Fusobacterium Nucleatum Stimulates Monocyte Adhesion to and Transmigration Through Endothelial Cells. Arch Oral Biol (2019) 100:86–92. 10.1016/j.archoralbio.2019.02.013 30818128

[25] Zha H Sun H Li X Duan L Li A Gu Y. S100A8 Facilitates the Migration of Colorectal Cancer Cells Through Regulating Macrophages in the Inflammatory Microenvironment. Oncol Rep (2016) 36(1):279–90. 10.3892/or.2016.4790 27176480

[26] Yu J Chen Y Fu X Zhou X Peng Y Shi L. Invasive Fusobacterium Nucleatum may Play a Role in the Carcinogenesis of Proximal Colon Cancer Through the Serrated Neoplasia Pathway. Int J Cancer (2016) 139(6):1318–26. 10.1002/ijc.30168 27130618

[27] Muller I Vogl T Pappritz K Miteva K Savvatis K Rohde D. Pathogenic Role of the Damage-Associated Molecular Patterns S100A8 and S100A9 in Coxsackievirus B3-Induced Myocarditis. Circ Heart Fail (2017) 10(11):e004125. 10.1161/CIRCHEARTFAILURE.117.004125 29158436

[28] Tsai SY Segovia JA Chang TH Morris IR Berton MT Tessier PA. DAMP Molecule S100A9 Acts as a Molecular Pattern to Enhance Inflammation During Influenza A Virus Infection: Role of DDX21-TRIF-TLR4-MyD88 Pathway. PLoS Pathog (2014) 10(1):e1003848. 10.1371/journal.ppat.1003848 24391503 PMC3879357

[29] Hold GL Garrett WS. Gut Microbiota. Microbiota Organization–a Key to Understanding CRC Development. Nat Rev Gastroenterol Hepatol (2015) 12(3):128–9. 10.1038/nrgastro.2015.25 25688055

[30] Larionova I Kazakova E Patysheva M Kzhyshkowska J. Transcriptional, Epigenetic and Metabolic Programming of Tumor-Associated Macrophages. Cancers (Basel) (2020) 12(6):1411. 10.3390/cancers12061411 PMC7352439 32486098

[31] Wang J Li D Cang H Guo B. Crosstalk Between Cancer and Immune Cells: Role of Tumor-Associated Macrophages in the Tumor Microenvironment. Cancer Med (2019) 8:4709–21. 10.1002/cam4.2327 PMC6712467 31222971

[32] Labonte AC Tosello-Trampont AC Hahn YS. The Role of Macrophage Polarization in Infectious and Inflammatory Diseases. Mol Cells (2014) 37(4):275–85. 10.14348/molcells.2014.2374 PMC4012075 24625576

[33] Mitsuhashi K Nosho K Sukawa Y Matsunaga Y Ito M Kurihara H. Association of Fusobacterium Species in Pancreatic Cancer Tissues With Molecular Features and Prognosis. Oncotarget (2015) 6(9):7209–20. 10.18632/oncotarget.3109 PMC4466679 25797243

[34] Yang NY Zhang Q Li JL Yang SH Shi Q. Progression of Periodontal Inflammation in Adolescents is Associated With Increased Number of Porphyromonas Gingivalis, Prevotella Intermedia, Tannerella Forsythensis, and Fusobacterium Nucleatum. Int J Paediatr Dent (2014) 24(3):226–33. 10.1111/ipd.12065 24025042

[35] Gur C Maalouf N Shhadeh A Berhani O Singer BB Bachrach G. Fusobacterium Nucleatum Supresses Anti-Tumor Immunity by Activating CEACAM1. Oncoimmunology (2019) 8(6):e1581531. 10.1080/2162402X.2019.1581531 31069151 PMC6492956

[36] Kostic AD Chun E Robertson L Glickman JN Gallini CA Michaud M. Fusobacterium Nucleatum Potentiates Intestinal Tumorigenesis and Modulates the Tumor-Immune Microenvironment. Cell Host Microbe (2013) 14(2):207–15. 10.1016/j.chom.2013.07.007 PMC3772512 23954159

[37] Hamada T Zhang X Mima K Bullman S Sukawa Y Nowak JA. Fusobacterium Nucleatum in Colorectal Cancer Relates to Immune Response Differentially by Tumor Microsatellite Instability Status. Cancer Immunol Res (2018) 6(11):1327–36. 10.1158/2326-6066.CIR-18-0174 PMC6215508 30228205

[38] Roth J Vogl T Sorg C Sunderkotter C. Phagocyte-Specific S100 Proteins: A Novel Group of Proinflammatory Molecules. Trends Immunol (2003) 24(4):155–8. 10.1016/s1471-4906(03)00062-0 12697438

[39] Kim JH Oh SH Kim EJ Park SJ Hong SP Cheon JH. The Role of Myofibroblasts in Upregulation of S100A8 and S100A9 and the Differentiation of Myeloid Cells in the Colorectal Cancer Microenvironment. Biochem Biophys Res Commun (2012) 423(1):60–6. 10.1016/j.bbrc.2012.05.081 22634002

[40] Hong W Khampang P Samuels TL Kerschner JE Yan K Simpson P. Expression of Calcium-Binding Proteins S100A8, S100A9 and S100A12 in Otitis Media. Int J Pediatr Otorhinolaryngol (2017) 101:30–6. 10.1016/j.ijporl.2017.07.025 28964306

[41] El-Shinawi M Mohamed HT El-Ghonaimy EA Tantawy M Younis A Schneider RJ. Human Cytomegalovirus Infection Enhances NF-kappaB/p65 Signaling in Inflammatory Breast Cancer Patients. PLoS One (2013) 8(2):e55755. 10.1371/journal.pone.0055755 23418456 PMC3572094

[42] Chan G Bivins-Smith ER Smith MS Yurochko AD. NF-Kappab and Phosphatidylinositol 3-Kinase Activity Mediates the HCMV-Induced Atypical M1/M2 Polarization of Monocytes. Virus Res (2009) 144(1-2):329–33. 10.1016/j.virusres.2009.04.026 PMC2736317 19427341

