# Supplementary material for: Fusobacterium nucleatum Facilitates M2 Macrophage Polarization and Colorectal Carcinoma Progression by Activating TLR4/NF-κB/S100A9 Cascade
Source: Front Immunol. 2021 May 21;12:658681. doi: 10.3389/fimmu.2021.658681 (PMC8176789; doi:10.3389/fimmu.2021.658681)
Supplement: Supplementary file 1 [file DataSheet_1.docx]

**Supplement figures**

**
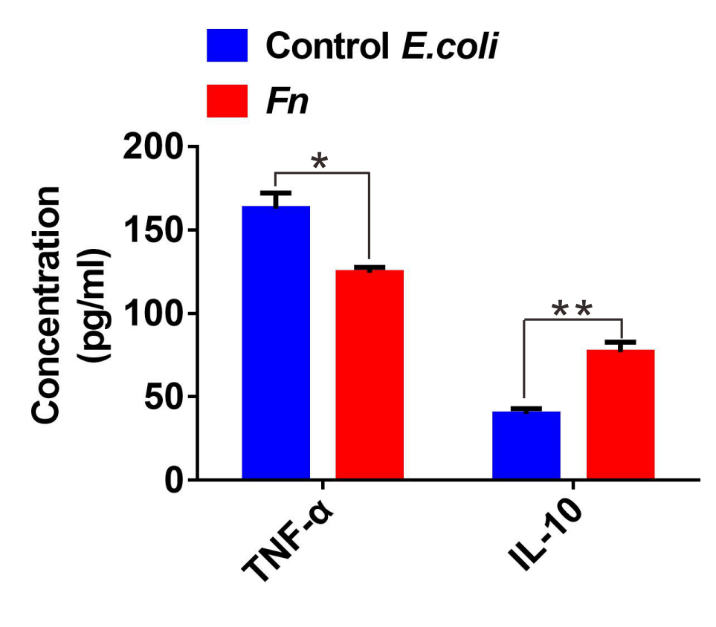
**

**Figure S1**. ELISA analysis of M1 marker (TNF-α) and M2 marker (IL-10) in cultured supernatant of macrophages treated with control *E. coli* or *Fn* for 48 h. Data were expressed as means ± SD in three independent experiments. ns: not significant. *p < 0.05, **p < 0.01.

**
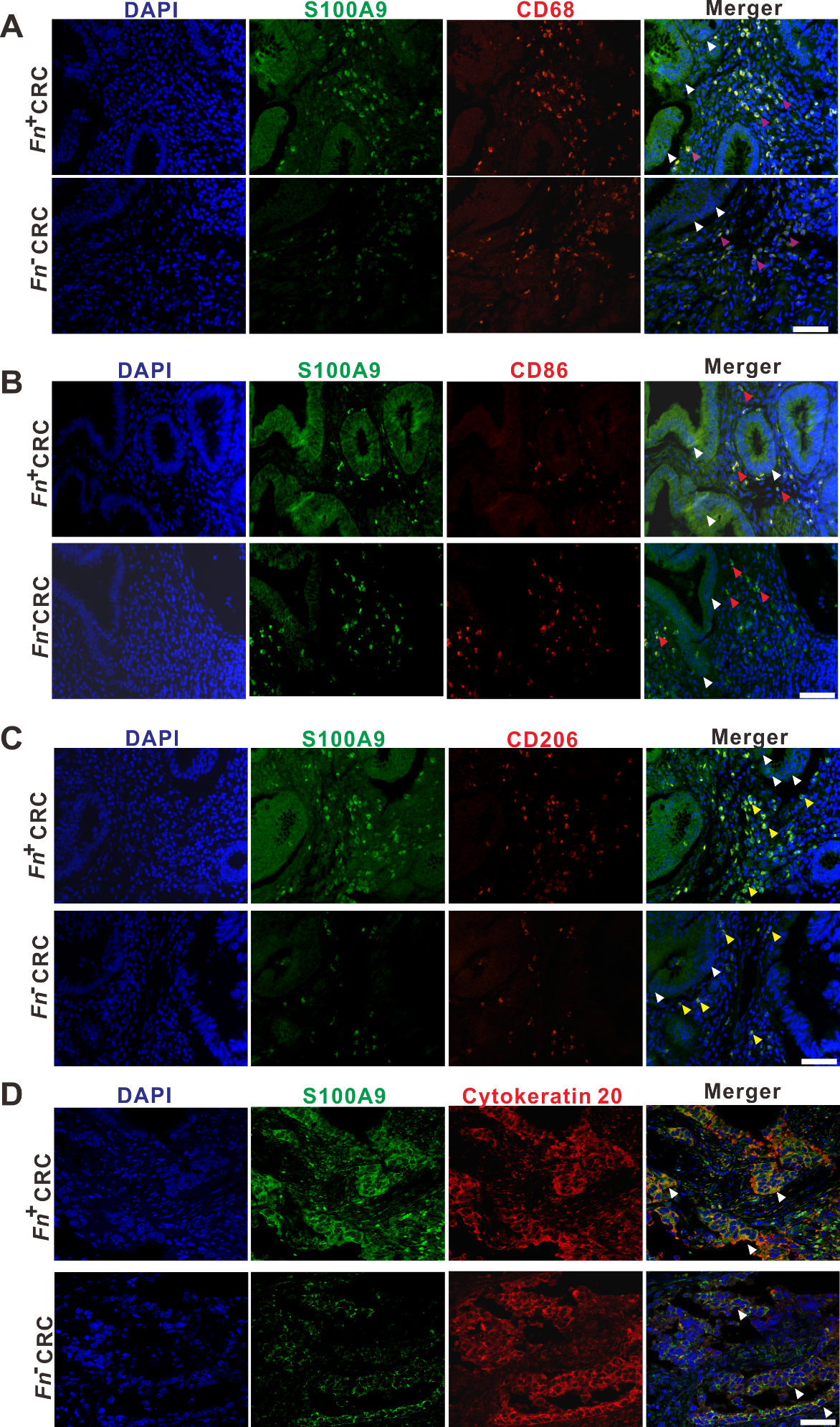
**

**Figure S2**. S100A9 levels in *Fn*-positive and *Fn*-negative CRC tissues. (B) Representative images of CD68 (red) and S100A9 (green) in tissues from *Fn*^+^ CRC and *Fn*^-^ CRC. S100A9 and CD68 were detected using speciﬁc antibodies. The ﬂuorescence patterns of the two proteins are overlapping. White arrow, CRC cells; Purple arrow, CD68^+^ M1-like Mφ. (B) Representative images of CD86 (red) and S100A9 (green) in tissues from *Fn*^+^ CRC and *Fn*^-^ CRC. S100A9 and CD86 were detected using speciﬁc antibodies. The ﬂuorescence patterns of the two proteins are overlapping. White arrow, CRC cells; Red arrow, CD86^+^ M1-like Mφ. (C) Representative images of CD206 (red) and S100A9 (green) in tissues from *Fn*^+^ CRC and *Fn*^-^ CRC. S100A9 and CD206 were detected using speciﬁc antibodies. The ﬂuorescence patterns of the two proteins are overlapping. White arrow, CRC cells; Yellow arrow, CD206^+^ M2-like Mφ. (D) Representative images of cytokeratin 20 (red) and S100A9 (green) in tissues from *Fn*^+^ CRC and *Fn*^-^ CRC. S100A9 and cytokeratin 20 were detected using speciﬁc antibodies. The ﬂuorescence patterns of the two proteins are overlapping. White arrow, CRC cells. White scale bars, 50 µm.

**
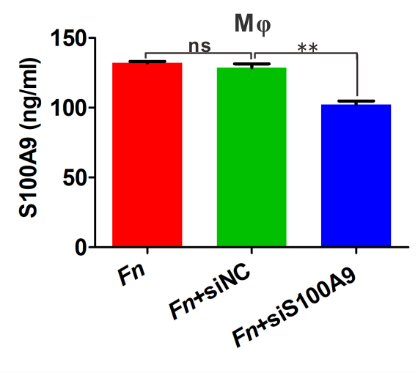
**

**Figure S3.** ELISA analysis of S100A9 level in the supernatant of Mφ transfected with siNC or siS100A9 and then co-cultured with *Fn*. Data shown are mean migrating cells±SD. ns: not significant. ***p* < 0.01.


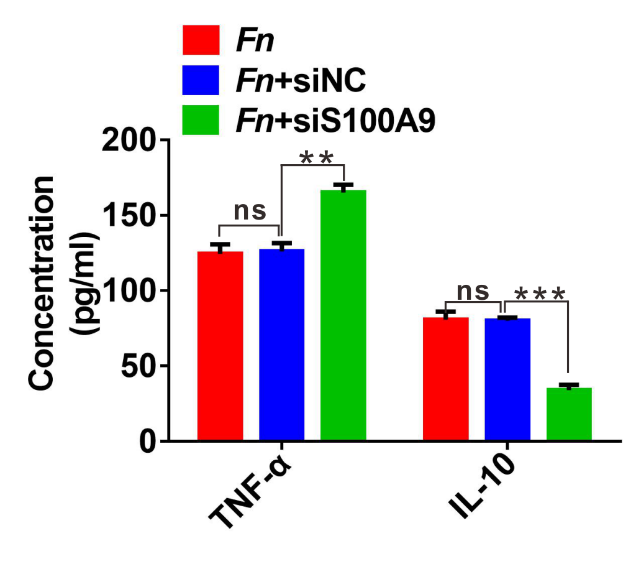


**Figure S4**. ELISA analysis of M1marker (TNF-α) and M2 marker (IL-10) in *Fn*-treated, (*Fn*+siNC)-treated and (*Fn*+siNC)-treated macrophages. Data were expressed as means ± SD in three independent experiments. ns: not significant. **p < 0.01, ***p < 0.001.


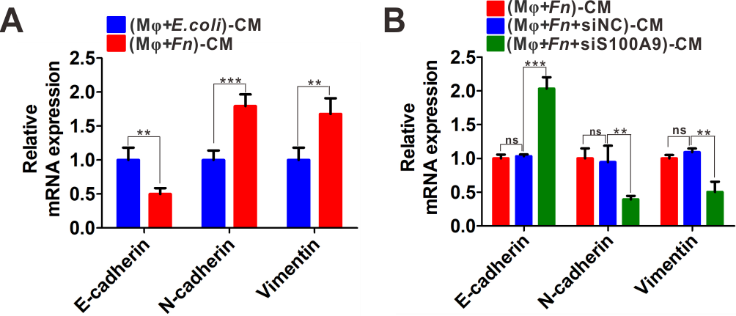


**Figure S5.** *Fn*-challenged M2-Mφ, which was mediated by S100A9, induces the change in gene levels of EMT-related biomarkers *E-cadherin*, *N-cadherin* and *Vimentin*. (**A)** qPCR analysis for genes levels of *E-cadherin*, *N-cadherin* and *Vimentin* in HCT116 cells cultured with (Mφ+*E.coli*)-CM and (Mφ+*Fn*)-CM for 24 h. (**B)** qPCR analysis for genes levels of *E-cadherin*, *N-cadherin* and *Vimentin* in HCT116 cells cultured with (Mφ+*Fn*)-CM, (Mφ+siNC+*Fn*)-CM and (Mφ+siS100A9+*Fn*)-CM for 24 h. Data shown are mean migrating cells±SD. ns: not significant. ***p* < 0.01, ****p* < 0.001.
