# Supplementary material for: Fusobacterium nucleatum Facilitates M2 Macrophage Polarization and Colorectal Carcinoma Progression by Activating TLR4/NF-κB/S100A9 Cascade
Source: Front Immunol. 2021 May 21;12:658681. doi: 10.3389/fimmu.2021.658681 (PMC8176789; doi:10.3389/fimmu.2021.658681)
Supplement: Supplementary file 2 [file Table_1.docx]

Table.S1 The clinicopathological parameters of collected samples.

| **Parameters** | **CRC** **(n=16)**  **n (%)** | **Distal normal tissues (n=16)**  **n (%)** |
| --- | --- | --- |
| **Gender** |  |  |
| Male (n, %) | 9 (56.25%) | 9 (56.25%) |
| Female (n, %) | 7 (43.75%) | 7 (43.75%) |
| **Age** |  |  |
| ＜60 (n, %) | 8 (50%) | 8 (50%) |
| ≥60 (n, %) | 8 (50%) | 8 (50%) |
| **Dukes staging** |  |  |
| A/B (n, %) | 6 (37.5%) | NA |
| C/D (n, %) | 10 (62.5%) | NA |
| **Lymphatic Metastasis** |  |  |
| Absent (n, %) | 6 (37.5%) | NA |
| Present (n, %) | 10 (62.5%) | NA |

NA, not available.
